# Clinical and self-reported markers of reproductive function in female survivors of childhood Hodgkin lymphoma

**DOI:** 10.1007/s00432-023-05035-z

**Published:** 2023-07-31

**Authors:** K. C. E. Drechsel, S. L. Broer, F. S. Stoutjesdijk, J. W. R. Twisk, M. H. van den Berg, C. B. Lambalk, F. E. van Leeuwen, A. Overbeek, M. M. van den Heuvel-Eibrink, W. van Dorp, A. C. H. de Vries, J. J. Loonen, H. J. van der Pal, L. C. Kremer, W. J. Tissing, B. Versluys, G. J. L. Kaspers, E. van Dulmen-den Broeder, M. A. Veening, the LATER-VEVO study group

**Affiliations:** 1 Emma Children’s Hospital, Amsterdam UMC, Vrije Universiteit Amsterdam, Pediatric Oncology, Amsterdam, The Netherlands; 2 Princess Máxima Center for Pediatric Oncology, Utrecht, The Netherlands; 3 Cancer Center Amsterdam, Amsterdam UMC, Location VUmc, Amsterdam, Netherlands; 4 Department of Reproductive Medicine & Gynecology, University Medical Center Utrecht, Utrecht, The Netherlands; 5 Department of Epidemiology and Data Science, Amsterdam UMC, Vrije Universiteit Amsterdam, Amsterdam, The Netherlands; 6 Department of Obstetrics and Gynaecology, Amsterdam UMC, Vrije Universiteit Amsterdam, Amsterdam, The Netherlands; 7 Department of Epidemiology, Netherlands Cancer Institute, Amsterdam, The Netherlands; 8 Department of Obstetrics and Gynaecology, Northwest Clinics, Alkmaar, The Netherlands; 9 Department of Paediatric Hemato-Oncology, Erasmus MC - Sophia Children’s Hospital, University Medical Center Rotterdam, Rotterdam, The Netherlands; 10 Division of Reproductive Endocrinology and Infertility, Department of Obstetrics and Gynecology, Erasmus University Medical Center, Rotterdam, The Netherlands; 11 Department of Hematology, Radboudumc Center of Expertise for Cancer Survivorship, Radboud University Medical Center, Nijmegen, The Netherlands; 12 Beatrix Children’s Hospital, University Medical Center Groningen, University of Groningen, Groningen, The Netherlands; 13 Department of Heamatology, Wilhelmina Children’s Hospital, University Medical Center Utrecht, Utrecht, The Netherlands

**Keywords:** Childhood Hodgkin lymphoma, Ovarian reserve, Anti-Mullerian hormone, Reproductive ability, Pregnancy

## Abstract

**Purpose:**

To evaluate the impact of treatment for Hodgkin lymphoma (HL) on clinical reproductive markers and pregnancy outcomes.

**Methods:**

This study was embedded within the DCOG LATER-VEVO study; a Dutch, multicenter, retrospective cohort study between 2004 and 2014. Serum anti-Müllerian hormone (AMH), follicle stimulating hormone (FSH), inhibin B, antral follicle count (AFC), and self-reported (first) pregnancy outcomes were evaluated in female childhood HL survivors and controls.

**Results:**

84 HL survivors and 798 controls were included, aged 29.6 and 32.7 years old at time of assessment. Median age at HL diagnosis was 13.4 years. Cyclophosphamide equivalent dose (CED-score) exceeded 6000 mg/m^2^ in 56 women and 14 survivors received pelvic irradiation.

All clinical markers were significantly deteriorated in survivors (odds-ratio for low AMH (< p10) 10.1 [95% CI 4.9; 20.6]; low AFC (< p10) 4.6 [95% CI 2.1; 9.9]; elevated FSH (> 10 IU/l) 15.3 [95% CI 5.7; 41.1], low Inhibin B (< 20 ng/l) 3.6 [ 95% CI 1.7; 7.7], p < 0.001). Pregnancy outcomes were comparable between survivors and controls (± 80% live birth, ± 20% miscarriage). However, survivors were significantly younger at first pregnancy (27.0 years vs 29.0 years, P = 0.04). Adjusted odds-ratio for time to pregnancy > 12 months was 2.5 [95% CI 1.1; 5.6] in survivors, p = 0.031. Adverse outcomes were specifically present after treatment with procarbazine and higher CED-score.

**Conclusion:**

HL survivors appear to have an impaired ovarian reserve. However, chance to achieve pregnancy seems reassuring at a young age. Additional follow-up studies are needed to assess fertile life span and reproductive potential of HL survivors, in particular for current HL treatments that are hypothesized to be less gonadotoxic.

**Supplementary Information:**

The online version contains supplementary material available at 10.1007/s00432-023-05035-z.

## Introduction

Hodgkin lymphoma (HL) accounts for approximately 5–6% of all childhood cancers (Siegel et al. 2023). Owing to improved treatment strategies, survival rates nowadays exceed 90% in pediatric patients (Borchmann et al. 2012; Mauz-Körholz et al. 2022). First-line HL treatment consists of consecutive multi-agent chemotherapy, with additional radiotherapy for some cases, depending on treatment response (Mauz-Körholz et al. 2022).

Both chemotherapy and radiotherapy are associated with late health effects, such as fertility issues that can substantially affect quality of life in survivorship (Landier et al. 2018; Robinson and Hudson 2014). With an increasing population of childhood cancer survivors (CCSs), there is a growing need to increase knowledge on the occurrence and, where possible, prevention of late effects of treatment.

Girls are born with a fixed pool of primordial follicles that progressively declines throughout life. The rate of oocyte quantity- and quality loss accelerates near the end of the fertile life, culminating in a fully depleted follicle pool at time of menopause at an average age of 51 years old (range 40–60 years old) (Broekmans et al. 2009; Morabia 1998; Te Velde and Pearson 2002). Cytotoxic treatments can induce apoptosis, which accelerates the natural process of oocyte depletion and could cause premature ovarian insufficiency (POI) (Bines et al. 1996; Chapman 1982; De Vos et al. 2010). POI is characterized by the combination of elevated gonadotropin levels, low estradiol levels and cycle irregularity (amenorrhea or oligomenorrhea) before the age of 40 years (De Vos et al. 2010; Webber et al. 2016). The decisive state of ovarian insufficiency is often preceded by incipient ovarian failure (IOF), during which fertility issues already tend to exist (Absolom et al. 2008).

Clinical markers to estimate the size of the remaining follicle pool include serum anti-Müllerian hormone (AMH) and the antral follicle count (AFC) measured by (transvaginal) ultrasound. Moreover, elevated serum follicle stimulating hormone (FSH) and low inhibin B levels can indicate impaired ovarian function, although levels significantly fluctuate throughout the menstrual cycle and tend to become abnormal relatively late in the process of follicle pool depletion (Broer et al. 2014; Parry and Koch 2019).

Studies among CSSs have shown that specific subgroups of survivors are at risk of having abnormal reproductive markers (low AMH, low AFC, high FSH) which may be a sign of fertility impairment or a reduced fertile life span (Charpentier et al. 2014; Krawczuk-Rybak et al. 2013; Roshandel et al. 2021; van den Berg et al. Van Den Berg et al. 2018, 2021). Moreover, several reports suggested reduced pregnancy rates, increased time to pregnancy and risk of premature delivery among CSSs (Anderson et al. 2018, 2022a, b; Armuand et al. 2017; Brämswig et al. 2015; Chow et al. 2016; Green et al. 2009; Madanat‐Harjuoja et al. 2010; Madanat et al. 2008; Magelssen et al. 2008; Oktem et al. 2018; Reulen et al. 2017; van de Loo et al. 2019; van Dijk et al. 2020). Adverse outcomes appear to specifically present after treatment with high doses of alkylating agents (particularly procarbazine) and abdominal/pelvic radiotherapy (van de Loo et al. 2019; Van Den Berg et al. 2018; van Dijk et al. 2020). These high-risk modalities have been essential components of HL treatment for decades, although current trials specifically aim to reduce and replace gonadotoxic modalities in standard HL treatment (European Network-Paediatric Hodgkin Lymphoma Study Group (EuroNet-PHL) 2015; Mauz-Körholz et al. 2022). Current evidence on fertility after treatment for HL mainly derives from cohort-studies including many different types of CCSs or adult (HL) survivors. Studies are small and heterogeneous, with a short follow up, and comparisons with a control population are sparse.

The aim of the current study was to assess the effect of childhood HL treatment on markers of ovarian reserve (AMH, AFC), ovarian function (FSH, inhibin B) and pregnancy rates and -outcomes (live birth, miscarriage, time to pregnancy, use of ART (assisted reproductive technology), premature delivery and having a small-for-gestational-age (SGA) infant). In addition, we aimed to identify treatment- and diagnosis related factors associated with impaired reproductive function.

## Methods

### Study design and study population

The present study is part of the Dutch Childhood Oncology Group (DCOG) LATER-VEVO study, a nationwide, multicenter, retrospective cohort study among Dutch female CCSs, that aimed to evaluate the effects of cancer treatment during childhood on reproductive function, ovarian reserve, and the risk of premature menopause. Details about the study design, the study population, and data collection methods, have been described previously (Overbeek et al. 2012; Van Den Berg et al. 2014, 2018). In short, the studied survivor-group consisted of adult women treated for cancer before the age of 18 between 1963 and 2002 who survived at least five years from diagnosis. The control group consisted of sisters from participating CCSs and women from the general population recruited through general practitioners’ offices. Control subjects had never been diagnosed with cancer.

In total 1106 female CCSs and 819 controls participated in the VEVO study. In the current study, only participating survivors treated for childhood HL were included, as well as controls. Survivors and controls who did not complete the questionnaire but only participated in the clinical part of the study were excluded from the analyses.

### Data collection and outcomes

Data were collected by questionnaire, blood sampling and a transvaginal ultrasound of the reproductive organs (Overbeek et al. 2012). Primary outcomes of the present study include (low) AMH and (low) AFC, while secondary outcomes comprise (high) FSH, (low) inhibin B and pregnancy rates- and outcomes.

*Questionnaire data* Baseline characteristics, including socio-demographic factors, smoking, weight and height, use of hormonal contraceptives, cycle characteristics and menopausal status were obtained by a questionnaire as was information on number of pregnancies, pregnancy outcomes, age at first birth, time to pregnancy (TTP, months of unprotected sexual intercourse until pregnancy) and method of conception (spontaneous versus using artificial reproductive technologies (ART) such as IUI or IVF/ICSI). Pregnancy outcomes included live birth, still birth (pregnancy loss after the 20th week of gestation), miscarriage (pregnancy loss before the 20th week of gestation), induced abortion (deliberate termination of pregnancy before the 24th week of gestation) and ectopic pregnancy. Live births before the 37th week of gestation were considered preterm and infants with birthweight below the 10th percentile of Dutch reference curves [PRN, (Visser et al. 2009)] were considered SGA. In this paper, only the results of the first attained pregnancy were assessed.

Educational levels up to and including lower technical and vocational training were categorised as ‘low’. Education up to and including secondary technical and vocational training was classified as ‘medium’ and educational levels up to and including higher technical and vocational training and university were classified as ‘high’. Menopause was defined as cessation of the menstrual cycle for at least 12 consecutive months, and menopause before the age of 40 was considered premature (Webber et al. 2016).

*Hormonal and ultrasound markers of reproductive function* Levels of FSH, AMH and Inhibin B were evaluated from collected serum samples, while AFC, defined as the number of all ovarian follicles sized 2-10 mm in both ovaries, was determined by transvaginal ultrasound. Blood sampling and ultrasound measurements were timed based on hormonal status, i.e. day 2–5 of a natural cycle or anytime in case of amenorrhea (no menses > 6 months). Females on hormonal contraceptives (HCs) were asked to discontinue HC use at least two months prior to study measurements. In females not wishing to temporarily cease HCs, measurements were planned on day 7 of the HC-free week.

Minimum detectable concentration for AMH was 29 pg/mL with an intermediate coefficient assay variation of 2.5% (ultra-sensitive immunoassay, pico-AMH, AnshLabs, USA). Lower limit of quantitation (LLOQ) for FSH was 0.5 IU/L, with 3–5% intra-assay variation and 6–7% inter-assay variation (immunometric assay, Delfia, Perkin Elmer, Wallac, Turku, Finland). Serum inhibin B levels were analyzed by the Gen II Inhibin B Elisa (Bechman Coulter), with LLOQ 11 ng/l and intra-assay and inter-assay variations of < 9% and < 10%, respectively (Van Den Berg et al. 2018).

Transvaginal ultrasounds were performed by trained personnel using a HD11 XE ultrasound system with 3D imaging. 3D data were analysed by two independent trained researchers, using customized software (provided by Philips Ultrasound, Inc.) and a pre-specified protocol. If the ovary was not found during the ultrasound evaluation, the AFC measurement (of that ovary) was imputed with ‘0’.

*Data on diagnosis and treatment* Data on (age at) HL diagnosis and received anti-cancer treatment were retrieved from (original) medical files. The cumulative alkylating agent exposure was estimated by calculating the cyclophosphamide equivalent dose (CED in mg/m^2^) (Green et al. 2014).

Age at time of study was determined based on date of birth and date of clinical assessment. For participants who did not participate in the clinical assessment, age at time of questionnaire was used.

### Statistical analysis

Missings in primary (AMH, AFC) or secondary outcomes (FSH, inhibin B, pregnancy rates- and outcomes) were listwise excluded. FSH serum levels that were reported as “ < 0.5” by the laboratory were replaced by 0.5 (1 HL survivor, 4 controls) to enable statistical analyses. Likewise, Inhibin B serum measurements “ < 10” were changed to 5.0 (10 HL survivors, 35 controls). Entries “0 months” and “0.5 months” for TTP within the questionnaire were corrected to 1 month (1 HL survivor and 13 controls).

AMH and AFC measurements below the p10 values of controls (determined by quantile regression analysis) were considered low. High FSH and low inhibin B were determined based on cut-off values (> 10 U/l for FSH and < 20 ng/l for Inhibin B, respectively) (Jiao et al. 2021; Laporte et al. 2011). Pregnancy outcomes were evaluated in the subgroup of females who stated they are currently attempting to become pregnant, ever had been pregnant or ever had tried to become pregnant (referred to as “ever attempted to become pregnant”). TTP was dichotomized during the analysis (i.e. < 12 months and > 12 months), as time to conceive exceeding a year was considered clinically relevant.

Baseline characteristics, clinical measurements, pregnancy rates and pregnancy outcomes were compared between HL survivors and controls. Results were presented as number (percentage) or median (IQR: interquartile range). Differences were analyzed using chi-square test or Fisher’s exact tests for categorical variables and Mann–Whitney U-test for continuous variables (because of non-normal distribution).

Moreover, clinical markers (i.e. AMH, FSH, Inhibin B and AFC) were evaluated in linear regression analyses and presented as beta-coefficient with 95% CI. The residuals of regression analyses of AMH values were non-normally distributed, and therefore, the regression model on AMH was performed on log-transformed values. The beta-coefficient was retransformed into the original scale and presented as Geometric Mean Ratio (GMR) with 95% confidence interval (CI).

Abnormal clinical markers (i.e. low AMH, high FSH, low Inhibin B and low AFC) and pregnancy rates and -outcomes were evaluated using logistic regression models. Results were expressed as odds-ratio with corresponding 95% CI. In a subgroup analysis, first pregnancy outcomes in HL survivors who received pelvic radiotherapy were compared to pregnancy outcomes in HL survivors who had no pelvic radiotherapy. A similar analysis was performed to compare HL survivors who had received treatment with CED score > 6000 mg/m^2^ and CED-score ≤ 6000 mg/m^2^. Regression models on hormonal and ultrasound data were adjusted for age at time of study, current smoking and current use of hormonal contraceptives. Analyses on overall pregnancy-, live birth- and miscarriage rates were adjusted for age at time of study, age at first pregnancy, educational level and marital status. The regression models on first pregnancy outcomes were adjusted for age at first pregnancy, educational level and marital status.

The contributing effects of diagnosis- and treatment-related factors on clinical markers were assessed using four different multivariable models, with each model evaluating a specific factor: (1) Age at time of diagnosis (< 10, 10–13, > 13 years), (2) Chemotherapy agents (cyclophosphamide, dacarbazine, procarbazine), (3) CED-score (0 mg/m^2^, 0–6000 mg/m^2^, > 6000 mg/m^2^) and (4) Radiotherapy body site (abdominal/pelvic area, other body sites, no radiotherapy). The control group was used as the reference group in all models. All models were adjusted for age at time of study, smoking and HC use. Depending on the factor of interest, additional corrections were added for pelvic radiotherapy (model 1, 2, 3) and/or CED score (model 1, 4).

All analyses were executed using R [version 4.0.3, (R Core Team 2018)]. P values < 0.05 were considered statistically significant.

## Results

### Included females

**Fig. 1:**
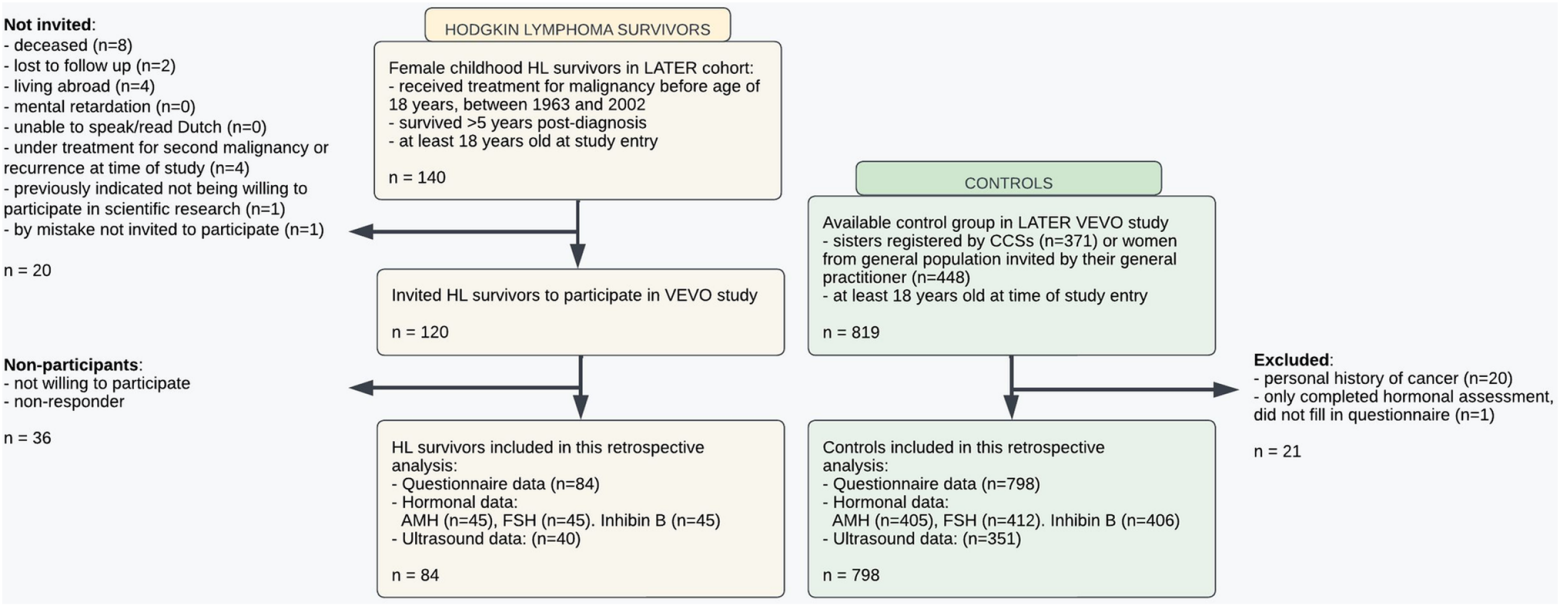
Selection process of female childhood Hodgkin lymphoma survivors and controls from the LATER VEVO study– cohort. *AMH* anti– Müllerian hormone, *CCSs* childhood cancer survivors, *FSH* Follicle stimulating hormone, *HL* Hodgkin lymphoma

A total of 84 HL survivors and 798 controls were included in the present study (see Fig. 1). Of these, 45 (53.5%) survivors and 413 (51.8%) controls provided a blood sample, while ultrasound data were available in 40 (47.6%) survivors and 351 (43.9%) controls, respectively. Questionnaires were completed between December 2004 and January 2014. Median interval between the date of questionnaire and date of clinical assessment was ± 1 month (range – 11; 25 months).

Baseline characteristics are presented in Table 1. Median age at time of study was 29.6 years (IQR 19.8; 51.4) in survivors and 32.7 years (IQR 19.7; 49.6) in controls (p = 0.08). Median BMI ranged from 22.6 to 23.0 kg/m^2^ (p = 0.47), most participants had medium to high educational levels (94.1–97.2%) and approximately 80% of both groups were married or in a relationship.

**Table 1:** Baseline characteristics of childhood Hodgkin lymphoma survivors and controls

	All included participants			Participants with available laboratory measurements or ultrasound data		
	HL survivor	Controls	P value	HL survivor	Controls	P value
	*n = 84*	*n = 798*		*n = 46*	*n = 415*	
**Age at time of study (years)**						
Median (IQR)	29.6 [19.8; 51.4]	32.7 [19.7; 49.6]	0.08	29.4 [20.7; 51.2]	32.8 [19.6; 52.1]	0.06
18–25	18 (21.4%)	163 (20.4%)	0.02	12 (26.1%)	85 (20.5%)	0.02
25–30	27 (32.1%)	146 (18.3%)		17 (37.0%)	79 (19.0%)	
30–35	16 (19.0%)	177 (22.2%)		5 (10.9%)	91 (21.9%)	
35–40	14 (16.7%)	144 (18.0%)		7 (15.2%)	66 (15.9%)	
≥ 40	9 (10.7%)	168 (21.1%)		5 (10.9%)	94 (22.7%)	
**Educational level (incl. current education)**^a^						
Low	5 (6.0%)	22 (2.8%)	0.11	3 (6.5%)	7 (1.7%)	0.13
Medium	35 (41.7%)	283 (35.8%)		17 (37.0%)	155 (37.6%)	
High	44 (52.4%)	486 (61.4%)		26 (56.5%)	250 (60.7%)	
**Marital status**						
Single	12 (14.3%)	141 (17.7%)	0.09	7 (15.2%)	94 (22.7%)	0.27
Married/relationship	66 (78.6%)	632 (79.5%)		36 (78.3%)	308 (74.2%)	
Divorced/widowed	6 (7.1%)	22 (2.8%)		3 (6.5%)	12 (2.9%)	
**BMI at time of study (kg/m**^2^)						
Median (IQR)	22.6 [18.1; 30.1]	23.0 [18.6; 36.8]	0.47	22.6 [18.0; 30.1]	23.0 [18.3; 37.6]	0.55
< 18.5	5 (6.0%)	16 (2.0%)	0.05	4 (8.7%)	11 (2.7%)	0.09
18.5–25	52 (62.7%)	530 (67.0%)		27 (58.7%)	269 (65.3%)	
25–30	22 (26.5%)	169 (21.4%)		13 (28.3%)	93 (22.3%)	
> 30	4 (4.8%)	76 (9.6%)		2 (4.3%)	40 (9.7%)	
**Current smoking**						
n (%)	9 (10.7%)	132 (16.5%)	0.42	8 (17.4%)	76 (18.3%)	1.000
**Menarchal age**						
Median (IQR)	13.0 [11.0; 16.0]	13.0 [10.0; 16.0]	0.29	13.0 [11.0; 16.0]	13.0 [10.0; 16.0]	0.59
**Cycle characteristics**						
Regular	28 (33.3%)	294 (37.4%)	0.25	15 (32.6%)	175 (42.7%)	0.55
Irregular	4 (4.8%)	26 (3.3%)		2 (4.3%)	16 (3.9%)	
No cycle, HC use	40 (47.6%)	399 (50.7%)		25 (54.3%)	192 (46.8%)	
No cycle, pregnant/breastfeeding	6 (7.1%)	46 (5.8%)		1 (2.2%)	12 (2.9%)	
Had ovariectomy	0 (0.0%)	1 (0.1%)		0 (0.0%)	0 (0.0%)	
Postmenopausal^c^	6 (7.1%)	21 (2.7%)		3 (6.5%)	15 (3.7%)	
**Timing clinical measurements**						
No clinical measurements available	38 (45.2%)	383(48.0%)	0.77	-	-	0.67
Cycle day 2–5	29 (34.5%)	254 (31.8%)		29 (63.0%)	254 (61.2%)	
Pill-free day 7	13 (15.5%)	135 (16.9%)		13 (28.3%)	135 (32.5%%)	
Anytime^d^	4 (4.8%)	26 (3.3%)		4 (8.7%)	26 (6.3%)	
**Age at diagnosis (years)**						
Median (IQR)	13.4 [6.4; 16.4]			13.7 [6.5; 15.9]		
< 10	13 (15.5%)			6 (13.0%)		
10–13	25 (29.8%)			11 (23.9%)		
> 13	46 (54.8%)			29 (63.0%)		
**Time since diagnosis (years)**						
Median (IQR)	16.5 [8.4; 36.6]			15.3 [8.3; 36.6]		
**Treatment era**						
1973–1980	10 (11.9%)			5 (10.9%)		
1980–1990	24 (28.6%)			10 (21.7%)		
1990–2001	50 (59.5%)			31 (67.4%)		
**Ann-arbor stage**						
1	8 (9.8%)			5 (8.9%)		
2	54 (65.9%)			33 (73.3%)		
3	16 (19.5%)			7 (15.6%)		
4	4 (4.9%)			1 (2.2%)		
Unknown	2 (2.4%)			0 (0.0%)		
**CT class**						
Alkylating agents	76 (93.8%)			42 (91.3%)		
Anthracyclines	69 (85.2%)			40 (87.0%)		
Epipodophyllotoxoin	10 (12.3%)			5 (10.9%)		
Vinca Alkaloids	80 (98.8%)			45 (97.8%)		
Platinum based CT	6 (7.4%)			3 (6.5%)		
Antimetabolites	7 (8.6%)			3 (6.5%)		
**Received CT agents**						
Bleomycine	50 (61.7%)			30 (65.2%)		
Carmustine	2 (2.5%)			0 (0%)		
Chloormetine	49 (60.5%)			28 (60.9%)		
Cisplatin	6 (7.4%)			3 (6.5%)		
Cyclophosphamide	17 (21.0%)			9 (19.6%)		
Cytarabine	7 (8.6%)			3 (6.5%)		
Dacarbazine	33 (40.7%)			19 (41.3%)		
Doxorubicine	61 (75.3%)			36 (78.3%)		
Epirubicine	8 (9.9%)			4 (8.7%)		
Etopside	10 (12.3%)			5 (10.9%)		
Ifosfamide	2 (2.5%)			1 (2.2%)		
Melphalan	1 (1.2%)			0 (0%)		
Methotrexaat	1 (1.2%)			1 (2.2%)		
Mitoxantrone	2 (2.5%)			2 (4.3%)		
Procarbazine	69 (85.2%)			39 (84.8%)		
Vinblastine	40 (49.4%)			23 (50.0%)		
Vincristine	74 (91.4%)			43 (93.5%)		
**Gonadotoxic risk of chemotherapeutic agents**^b^						
High risk	71 (87.7%)			40 (87.0%)		
Medium risk	7 (8.6%)			5 (10.9%)		
Low or no risk	0 (0.0%)			0 (0%)		
Unknown risk	3 (3.7%)			1 (2.2%)		
No chemotherapy	3 (3.6%)			0 (0%)		
**CED-score (mg/ m**^2^)						
0	10 (11.9%)			6 (13.0%)		
0–6000	14 (16.7%)			10 (21.7%)		
> 6000	56 (66.7%)			29 (63.0%)		
**Radiotherapy site**^e^						
No radiotherapy	37 (44.0%)			22 (47.8%)		
Cranial/spinal	35 (41.6%)			20 (43.5%)		
Abdominal/pelvic	14 (16.7%)			5 (10.9%)		
TBI	0 (0.0%)			0 (0%)		
Other^f^	40 (47.6%)			19 (41.3%)		
**Stem cell transplantation**						
Allogenic	0 (0.0%)			0 (0%)		
Autologous	2 (2.4%)			0 (0%)		

*IQR* Interquartile range, *BMI* body mass index, *TBI* total body irradiation, *CT* chemotherapy, *RT* radiotherapy, *CED* cyclophosphamide equivalent dose

Values represent the number (%) of women, unless indicated otherwise. The subcategories may not add up to the total number of women due to missing values

^a^Categorized as low: up to and including lower technical and vocational training; medium: up to and including secondary technical and vocational training; high: up to and including higher technical and vocational training and university

^b^Risk classification (adapted from Kim and Jeon 2012; Rodriguez-Wallberg et al. 2020) A) High risk: Cyclophosphamide, cyclofosfamide, melphalan, procarbazine, ifosfamide. B) Intermediate risk: Cisplatin, Adriamycin = doxorubicine. C) Unknown risk: Dacarbazine. D) Low risk: Bleomycine, methotrexate, vincristine

^c^n = 1 (1.2%) HL survivor and n = 0 (0%) control, p = 0.182 experienced non-surgical premature menopause (before the age of 40 years)

^d^Anytime, due to amenorrhea, because of postmenopausal status (n = 3 HL survivors, n = 15 controls) or cycle not yet recognizable after pregnancy (n = 1 HL survivors, n = 11 controls)

^e^Numbers will not add up as survivors may have received radiotherapy at multiple sites

^f^Other radiated areas include thorax (n = 37), upper extremities (n = 1), thorax and upper extremities (n = 1) or unknown (n = 1)

There were no statistically significant differences in menarcheal age or cycle characteristics between the two groups of interest. There were 27 postmenopausal women included in this study (6 (7.1%) survivors and 21 (2.7%) controls). Of the menopausal survivors, only 1 (1.2%) was diagnosed with POI. However, only 9 survivors (10.7%) and 168 controls (21.1%) were aged > 40 years at time of the study.

In total, 40 survivors (47.6) and 399 controls (50.7%) used a form of hormonal contraceptives at time of study invitation. Approximately 50% of these women did not participate in the clinical part of the study. Timing of clinical measurements of the other 50% is reported in Table 1. There were no statistically significant differences in use of hormonal contraceptives and timing of clinical measurements between survivors and controls.

Among the survivors, median age at HL diagnosis was 13.4 years old (IQR 6.4; 16.4), with a median time since diagnosis of 16.5 years (IQR 8.4; 36.6) (Table 1). Ann Arbor stage was 1 or 2 in approximately 75% of cases. Many women (n = 71, 87.7%) received chemotherapeutic agents that are commonly considered as high risk for gonadotoxicity (i.e. cyclophosphamide, melphalan, procarbazine and/or ifosfamide). CED-score exceeded 6000 mg/m^2^ in 56 (66.7%) women. Three survivors (3.6%) did not receive any chemotherapy and were treated locally with radiotherapy only. In total, 47 women (56.0%) received radiotherapy, of whom 14 (16.7%) were irradiated to the abdominal/pelvic area (20-40 Gy). Two women (2.4%) received autologous stem cell transplantation.

Baseline characteristics of the subset of survivors and controls in whom laboratory measurements and/or ultrasound data were available are described in separate columns in Table 1**.** There were no substantial differences in baseline data, when compared to all included participants.

### Clinical measurements

#### Anti-mullerian hormone and antral follicle count

Unadjusted median AMH serum levels were 0.5 ng/ml (IQR < 0.1; 7.5) in 45 survivors and 1.8 ng/ml (IQR < 0.1; 11.8) in 403 controls (p < 0.001), see Table 2. Adjusted GMR was 0.6 (95% CI 0.6; 0.7; *p* < 0.001).

**Table 2:** Hormonal and ultrasound markers of reproductive potential in childhood Hodgkin lymphoma survivors and controls

	HL survivors	Controls	P value
**AMH (ng/ml)**	*n = 45*	*n = 405*	
Median (IQR)	0.5 [< 0.1; 7.5]	1.8 [< 0.1; 11.7]	< 0.001
GMR* (95% CI)	0.6 [0.6; 0.7]	Ref	< 0.001
**Low AMH (< p10)**^a^			
n (%)	21 (46.7%)	32 (7.9%)	< 0.001
Odds-ratio (95% CI)	10.1 [4.9–20.6]	Ref	< 0.001
**FSH (U/L)**	*n = 45*	*n = 412*	
Median (IQR)	7.7 [1.8; 51.9]	5.9 [1.8; 55.7]	0.006
β (95% CI)	5.0 [1.7; 8.4]	Ref	0.003
**Elevated FSH (≥ 10 U/L)**			
n (%)	16 (35.6%)	50 (12.1%)	< 0.001
Odds-ratio (95% CI)	15.3 [5.7; 41.1]	Ref	< 0.001
**Inhibin B (ng/L)**	*n = 45*	*n = 406*	
Median (IQR)	50.1 [5.0; 123.0]	70.2 [5.0; 183.7]	0.01
β (95% CI)	– 22.6 [– 36.7; – 8.4]	Ref	0.002
**Decreased Inhibin-B (≤ 20 ng/L)**			
n (%)	15 (33.3%)	68 (16.7%)	0.01
Odds-ratio (95% CI)	3.6 [1.7–7.7]	Ref	0.001
**AFC**	*n = 40*	*n = 351*	
Median (IQR)	11.0 [0.0; 26.3]	15.0 [0.8; 48.5]	0.02
β (95% CI)	– 6.9 [– 10.2; – 3.7]	Ref	< 0.001
**Low AFC (< p10)**^a^			
n (%)	14 (35.0%)	37 (10.5%)	< 0.001
Odds-ratio (95% CI)	4.6 [2.1; 9.9]	Ref	< 0.001

*AMH* anti-Mullerian hormone, *FSH* follicle stimulating hormone, *AFC* antral follicle count, *GMR* Geometric Mean Ratio, *SD* standard deviation, *CT* chemotherapy, *RT* radiotherapy, *n* number

P-values for median values and numbers are calculated using Fisher’s exact/Chi-square or Mann–Whitney U

All regression analyses were adjusted for Age at time of study, current smoking and current use of hormonal contraceptives

*Geometric mean ratio (GMR) was calculated by back-transforming (exp(b) the regression coefficient, performed on log-transformed AMH values

^a^AMH measurements were considered as < p10 if values were below 1.953 + -0.043*Age. AFC measurements were considered as < p10 if values were below 23.379 + – 0.498*Age. Both equations are based on quantile regression analysis in the control group (AMH n = 405 controls; AFC n = 351 controls, respectively)

**Fig. 2:**
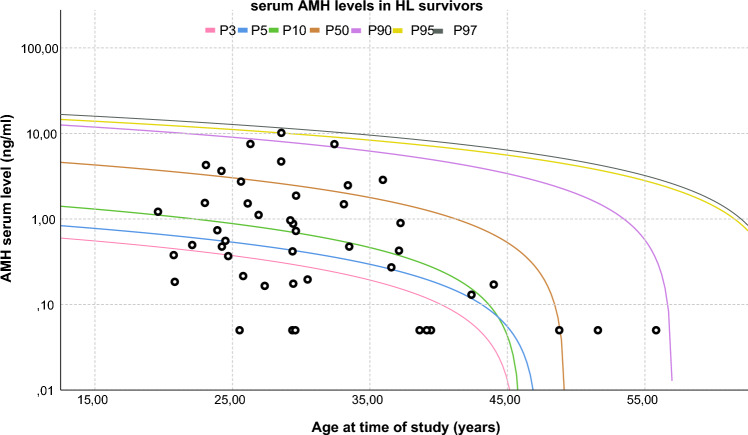
Scatterplot of anti– Müllerian hormone (AMH) measurements in Hodgkin lymphoma survivors. *AMH* anti– Müllerian hormone, *HL* Hodgkin lymphoma. Scatterplot including all AMH serum measurements in the included HL survivors (n = 45). Each black dot represents a single measurement. Depicted reference lines are based on parameter estimates by quantile regression analysis in the control group (n = 406 AMH measurements) P3: − 0.021* age + 0.994; P5: − 0.024 * age + 1.135; P10: − 0.043 * age + 1.953; P50: − 0.125 * age + 6.155; P90: − 0.282 * age + 16.079; P95: − 0.277 * age + 18.040; P97: − 0.317 * age + 20.652

In Fig. 2 all individual AMH serum measurements in survivors are depicted in a scatterplot. Included reference lines represent percentile lines of the measurements in the control group. There were 21 (46.7%) survivors with low AMH serum levels (< p10 of controls). This proportion was statistically significantly higher in the survivor group, when compared to the control group (32 (7.9%) controls with AMH < p10; p < 0.001). Adjusted odds for having a low AMH as a survivor was 10.1 (95% CI 4.9; 20.6; p < 0.001).

In total, 393 women (40 survivors, 353 controls) underwent a vaginal ultrasound. Two assessments were excluded from the analyses because of ovariectomy (2 controls). Median AFC was 11.0 (IQR 0.0; 26.3) in survivors and 15.0 (IQR 0.8; 48.5) in controls. A significant effect of HL survivorship on the AFC measurement was seen in adjusted regression analysis, β – 6.9 (95% CI – 10.2; – 3.7; p =  < 0.001). A total of 14 (35.0%) survivors had low AFC measurements (< p10 of controls). The adjusted odds-ratio for having a low AFC as a survivor was 4.6 (95% CI 2.1; 9.9; p < 0.001). Results are also presented in Table 2.

#### Follicle stimulating hormone and inhibin B

As reported in Table 2, median FSH levels were significantly higher, and Inhibin B levels were significantly lower in the survivor group, when compared to controls (FSH levels: 7.7 U/l (IQR 1.8; 51.9) in survivors, 5.9 U/l (IQR 1.8; 55.9) in controls; p = 0.006 and Inhibin B levels: 50.1 ng/l (IQR 5.0; 123; 0) in survivors, 70.4 ng/l (5.0; 183.9) in controls; p = 0.010, respectively). Adjusted analyses resulted in similarly significant results. Roughly 30% of the HL survivors had high FSH (≥ 10 U/l) levels and/or decreased inhibin B levels (≤ 20 ng/l), compared to 10–15% of controls. Odds ratios for having these abnormal markers of gonadal function as a survivor were 15.3 (95% CI 5.7; 41.1) in case of elevated FSH and 3.6 (95% CI 1.7; 7.7) in case of decreased inhibin B (both p < 0.001).

#### Consistency in clinical markers of reproductive function

Data on all included clinical markers were available in 39 survivors and 340 controls. In 46% (18) of survivors and 74% (253) of controls, AMH, AFC, FSH and Inhibin B levels were all normal. All markers were abnormal in 8 (20.5%) survivors and 4 (1.2%) controls. Six (15.4%) survivors and 9 (2.6%) controls only had low AMH levels, while the other measurements were still within normal range (see Supplementary file 1).

### Time to (first) pregnancy and pregnancy outcomes

Of all participating women, 47 (56.0%) survivors and 430 (53.9%) controls stated in the questionnaire that they had (ever) tried to become pregnant. Women who did not attempt pregnancy were mainly young (74.7% of survivors and 69.3% of controls were below age of 30 years) and reported that there were circumstances currently preventing them to attempt a pregnancy (see Online Resource 2). In both groups, approximately 92% of the women who ever attempted to become pregnant succeeded, 81% ever achieved a live birth and 21% ever experienced a miscarriage. There were no statistically significant differences between survivors and controls.

Forty-three survivors (100%) and 389 out of 396 controls (98%) reported details on their first pregnancy (Table 3). Median age of survivors at first pregnancy was 2 years younger when compared to controls (27.0 years old (IQR 20.0; 35.0) versus 29.0 years (IQR 18.0; 37.0); p = 0.04, respectively). Median interval between HL diagnosis and first pregnancy was 13.8 years (IQR 3.1; 24.8). Most females conceived spontaneously (95.4% of survivors and 94.6% of controls).

**Table 3:** First pregnancy in childhood Hodgkin lymphoma survivors and controls

	HL survivors	Controls	P value
*All women*	*n = 84*	*n = 798*	
**Ever attempted to become pregnant**^a^			
n (%)	47 (56.0%)	430 (53.9%)	0.81
Odds-ratio (95% CI)	1.4 [0.7–2.6]	Ref	0.23
*Women who attempted to become pregnant*	*n = 47*	*n = 430*	
**Ever pregnant**			
n (%)	43 (91.5%)	396 (92.1%)	0.78
Odds-ratio (95% CI)	1.1 [0.4; 3.4]	Ref	0.85
**Ever achieved a live birth**			
n (%)	38 (80.9%)	349 (81.2%)	1.000
Odds-ratio (95% CI)	1.3 [0.6; 3.0]	Ref	0.52
**Ever miscarried**			
n (%)	10 (21.3%)	94 (21.9%)	1.000
Odds-ratio (95% CI)	1.1 [0.5–2.4]	Ref	0.75
*Outcomes for (first) pregnancies within cohort*	*n* = *43*	*n* = *389*	
**Age at first pregnancy (years)**			
Median (IQR)	27.0 [20.0; 35.0]	29.0 [18.0; 37.0]	0.04
**Currently pregnant (first pregnancy)**			
n (%)	3 (7.0%)	13 (3.3%)	0.21
**Live birth**			
n (%)	34 (79.1%)	296 (76.1%)	0.81
Odds-ratio (95% CI)	1.3 [0.6; 2.8]	Ref	0.58
**Still birth**			
n (%)	0 (0.0%)	3 (0.8%)	1.000
Odds-ratio (95% CI)	0.0 [0.0; inf]	Ref	1.000
**Miscarriage**			
n (%)	3 (7.0%)	34 (8.7%)	1.000
Odds-ratio (95% CI)	0.9 [0.2; 3.0]	Ref	0.79
**Induced abortion**			
n (%)	3 (7.0%)	40 (10.3%)	0.79
Odds-ratio (95% CI)	0.6 [0.2; 2.2]	Ref	0.43
**Ectopic pregnancy**			
n (%)	0 (0.0%)	4 (1.0%)	1.000
Odds-ratio (95% CI)	0.0 [0.0-inf]	Ref	0.99
**Time to pregnancy (TTP) (months)**			
Median (IQR)	4.5 [1.0; 24.0]	3.0 [1.0; 38.8]	0.15
n (%) with TTP > 12 months	10 (23.8%)	45 (12.4%)	0.07
Odds-ratio (95% CI), TTP > 12 months	2.3 [1.0; 5.2]	Ref	0.04
**Use of ART**			
n (%)	2 (4.6%)	21 (5.4%)	1.000
IUI	1 (2.3%)	12 (3.1%)	
IVF/ICSI	1 (2.3%)	9 (2.3%)	
Odds-ratio (95% CI), use of ART	1.0 [0.2; 4.8]	Ref	1.000
*Obstetric outcomes*	*n = 43*	*n = 389*	
**Birth weight (gram)**			
Median (IQR)	3240.0 [1591.0; 4392.8]	3369.0 [2051.1; 4460.4]	0.42
**Small for gestational age (SGA)**			
n (%)	4 (12.1%)	50 (17.1%)	0.63
Odds-ratio (95% CI)	0.6 [0.2; 1.8]	Ref	0.33
**GA at delivery**			
Median (IQR)	[31.6; 42.0]	40.0 [34.0; 42.0]	0.08
**Preterm delivery**			
n (%)	3 (8.8%)	21 (7.1%)	0.72
Odds-ratio (95% CI)	1.1 [0.3; 3.8]	Ref	0.95

*GA* gestational age, *ART* artificial reproductive techniques, *IUI* intra-uterine insemination, *IVF* in-vitro fertilization, *ICSI* intracytoplasmatic sperm injection, *TTP* time to pregnancy

^a^Females who stated they do not have an (active) child wish, only have a future child wish or do not (yet) know if they wish to have children, were classified as “never attempting to become pregnant”. Females who stated they are currently attempting to become pregnant, ever been pregnant or ever tried to become pregnant, were classified as “attempting to become pregnant”

Regression analyses on “ever attempted to become pregnant”, “ever pregnant”, “ever achieved a live birth” and “ever miscarried” were adjusted for Age at time of study, Age at first pregnancy, educational level and marital status. Regression models on first pregnancy outcomes were adjusted for Age at first pregnancy, educational level and marital status. n = 26 missings in TTP, n = 105 missings in SGA and n = 101 missings in preterm delivery were excluded from regression analyses

There was no difference in use of ART among the two studied groups (4.6% in survivors versus 5.4% in controls; p = 1.000, respectively). Time to pregnancy was 4.5 months (IQR 1.0; 24.0) in survivors and 3.0 months (IQR 1.0; 38.8) in controls (p = 0.15).

The number of women with a time to pregnancy over a year appeared to be higher in HL survivors (n = 10, 23.8%), when compared to controls (n = 45, 2.4%) (p = 0.07), but only the adjusted odds-ratio for TTP > 12 months reached statistical significance (odds-ratio 2.5 (95% CI 1.1; 5.6); p = 0.03). These survivors had a median age at first pregnancy of 29.5 years (IQR 21.5; 37.2), CED-score exceeded 6000 mg/m^2^ in 8 (80%) women and 4 (40%) had received pelvic radiotherapy.

When looking at the pregnancy outcomes in the survivor- and control group, no statistically significant differences were observed in live birth-, still birth-, miscarriage-, induced abortion- and ectopic pregnancy rates. Median birthweight and gestational age at delivery were comparable. In addition, no statistically significantly differences were observed in pregnancy rates and -outcomes when comparing HL survivors based on whether they received pelvic radiotherapy (n = 10 ‘yes’, n = 33 ‘no’) or the CED-score of received treatment (n = 9 ‘ ≤ 6000 mg/m^2^’, n = 32’ > 6000 mg/m^2^’) (see Online Resource 3 and 4). However, all survivors who experienced a preterm birth (n = 3) or gave birth to an SGA infant (n = 4) had received high-risk treatment with CED > 6000 mg/m^2^, p = 0.566 and p = 1.000.

### Diagnosis and treatment related factors

Results of the regression models including diagnosis- and treatment related factors are included in Table 4. Overall, the deteriorating effect of treatment on reproductive markers appeared to be specifically present in protocols with procarbazine (GMR AMH 0.4 (95% CI 0.3; 0.6 95% CI); p < 0.001, B-coefficient AFC – 9.2 (95% CI – 17.9; – 0.4); p = 0.04). A higher CED-score had a negative effect on all clinical markers. There was no such pronounced effect of (pelvic) radiotherapy on the assessed measurements, although there was a trend towards the most abnormal values of the clinical markers in the subset of patients receiving pelvic radiotherapy. No clear effect of age at diagnosis was observed.

**Table 4:** Diagnosis and treatment-related factors associated with abnormal markers of reproductive potential in childhood Hodgkin lymphoma survivors

	Hormonal data							Ultrasounddata		
	AMH			FSH		Inhibin– B		AFC		
	n	GMR (95% CI)	P value	B-coefficient (95% CI)	P value	B-coefficient (95% CI)	P value	n	B-coefficient (95% CI)	P value
**Age at diagnosis (years)**										
< 10	6	0.9 [0.3; 2.6]	0.883	10.4 [– 13.0; 33.8]	0.381	– 30.7 [– 130.6; 69.3]	0.547	6	– 8.8 [– 17.0; – 0.5]	0.04
10–13	10	0.8 [0.3; 2.3]	0.731	9.8 [– 12.8; 32.5]	0.394	– 30.3 [– 127.1; 66.5]	0.539	8	– 9.6 [– 16.9; – 2.3]	0.01
> 13	29	0.7 [0.3; 1.7]	0.420	14.7 [– 6.6; 36.0]	0.175	– 35.2 [– 126.2; 55.9]	0.448	26	– 8.6 [– 13.6; – 3.7]	0.001
**Chemotherapy agents**										
Cyclophosphamide	9	1.0 [0.6; 1.6]	0.92	0.8 [– 10.3; 11.6]	0.92	– 19.0 [– 65.2; 27.2]	0.42	6	– 2.7 [– 14.7; 9.3]	0.66
Dacarbazine	19	1.2 [0.9; 1.7]	0.12	– 8.2 [– 15.6; – 0.9]	0.03	24.9 [– 6.3; 56.2]	0.12	17	0.7 [– 6.1; 7.5]	0.85
Procarbazine	38	0.4 [0.3; 0.6]	< 0.001	6.7 [– 2.3; 15.7]	0.14	– 5.4 [– 43.6; 32.8]	0.78	34	– 9.2 [– 17.9; – 0.4]	0.04
**CED score (mg/ m2)**										
0	6	1.4 [1.0; 2.1]	0.07	– 1.5 [– 10.2; 7.2]	0.74	– 19.3 [– 56.4; 17.8]	0.31	6	1.7 [– 6.2; 9.6]	0.67
> 0–6000	9	0.7 [0.5; 0.9]	0.01	3.0 [– 4.2; 10.2]	0.41	– 13.4 [– 44.1; 17.4]	0.39	9	– 5.4 [– 11.9; 1.2]	0.11
> 6000	29	0.5 [0.4; 0.6]	< 0.001	6.4 [2.1; 10.7]	0.004	– 23.5 [– 41.9; – 5.2]	0.01	25	– 8.9 [– 13.0; – 4.7]	< 0.001
**Radiotherapy body site**										
Abdominal/pelvic area included	5	0.4 [0.2; 1.22]	0.11	16.0 [– 7.9; 39.9]	0.19	– 51.5 [– 153.4; 50.4]	0.32	4	– 13.3 [– 23.1; – 3.5]	0.008
Only other sites	19	0.7 [0.3; 1.8]	0.45	14.2 [8.3; 36.6]	0.22	– 43.8 [– 139.5; 52.0]	0.37	16	– 9.3 [– 15.1; – 3.4]	0.002
No radiotherapy	21	0.7 [0.3; 1.7]	0.43	14.6 [– 6.7; 35.9]	0.18	– 35.1 [– 126.0; 55.8]	0.45	20	– 8.6 [– 13.4; – 3.8]	< 0.001

*AMH* anti– Mullerian hormone, *AFC* antral follicle count, *CED* cyclophosphamide equivalent dose, *CT* chemotherapy, *FSH* follicle stimulating hormone, *GMR* geometric mean ratio, *RT* radiotherapy, *n* number

AMH measurements were considered as < p10 if values were below 1.953 + − 0.043*Age. AFC measurements were considered as < p10 if values were below 27.475 + -0.594*Age. Both equations are based on quantile regression analysis in the control group (AMH n = 403 controls; AFC n = 332 controls, respectively)

All models were adjusted for age at time of study, smoking and HC use. Depending on the factor of interest, additional corrections were added for pelvic radiotherapy (model 1: “age at diagnosis”, 2: “chemotherapy” and 3: “CED score”) and/or CED score (model 1: “age at diagnosis”, 4: “Radiotherapy body site”)

Controls were used as the reference group in all models

## Discussion

This study broadly evaluated (indirect) clinical markers of ovarian reserve and -function, and (first) pregnancy results in a group of childhood HL survivors, compared to siblings and controls from the general population. All assessed clinical markers, including serum AMH, FSH, inhibin B and AFC measured by ultrasound, were significantly worse in survivors compared to controls. Survivors had a lower age at first pregnancy and a longer time to first pregnancy (> 12 months). However, when they did become pregnant, pregnancy outcome and live birth rates were comparable between the survivor and the control group.

### Ovarian reserve and ovarian function

HL survivors were more likely to have lower AMH serum levels and AFC measurements, when compared to the control population. Almost half of the 45 assessed survivors in this study had AMH levels below p10 of healthy controls. Two previous cross-sectional studies on female HL survivors reported comparable results, even though applied cut-off values to define low AMH varied (Beek et al. 2007; Charpentier et al. 2014). The age-dependent changes of AMH serum levels should be considered when determining the cut-off value to classify ‘low’ levels (Fleming et al. 2012; Kelsey et al. 2011). In this study, the cut-off value of p10 was established to identify survivors at risk of a shortened reproductive life span. This cutoff value is relatively conservative and may have resulted in an over-estimation of low AMH in this cohort. However, the timely recognition of survivors at risk of impaired fertility is crucial in order to be able to act upon it, i.e. survivors could be advised to not postpone an active childwish or preserve fertility during survivorship.

During and directly after cancer treatment, a significant drop in AMH is often seen (Anderson, et al. 2022a, b). Subsequent recovery may occur. Previous studies reported peak concentrations of AMH 2–3 years post-treatment followed by a continuous, gradual decline (Irene Su et al. 2020). The present study included HL survivors with a median time of 16.5 years (IQR 8.4; 36.6) since diagnosis, hence additional improvement or recovery of serum AMH seems highly unlikely in this cohort. A longitudinal follow-up study among female CCSs demonstrated that the pattern of AMH decrease over a 3-year period (median 16 years post-treatment) was consistent with the pattern of AMH decline observed in healthy women (Van Der Kooi et al. 2017). These results suggest that the follicle reserve pool could be reduced after gonadotoxic cancer treatment, while the process of oocyte decay (of the remaining follicle pool) may not necessarily be accelerated during survivorship (Anderson and Su 2020).

High FSH serum levels and decreased inhibin B concentrations, as seen in the survivor group, are associated with impaired reproductive function. Most women who did not use hormonal contraceptives had regular cycles. One of the survivors had experienced premature menopause, with a group prevalence of 1%. However, only 9 out of the 84 survivors had reached the age of 40 years at time of the questionnaire, and premature deterioration of ovarian function could still occur.

### Pregnancy

Despite significant differences in clinical markers between survivors and controls, not in favor of survivors, the reported overall live birth rate and miscarriage rates were similar in both groups (~80% and ~20% in both the survivor- and control-group). These rates are very similar to pregnancy outcomes in the general population (Gnoth et al. 2003; Wang et al. 2003; Wesselink et al. 2017). Most previous (cohort)studies mentioned number of female HL survivors achieving pregnancy or a live birth during follow up, without evaluating their wish to conceive, time to conceive or other fertility-related factors that may affect their reproductive potential (Beek et al. 2007; Drechsel et al. 2023; Fernandez-Pineda et al. 2018; Gözdasoglu et al. 1995; Green and Hall 1988; Horning et al. 1981; Licht et al. 2021; Mackie et al. 1996; Madsen et al. 1995; McCullough et al. 2010; Papadakis et al. 1999; Perrone et al. 1989; Reulen et al. 2009; Swerdlow et al. 1996; Sy Ortin et al. 1990; van der Kaaij et al. 2012; Wilimas et al. 1980). Among the two studies who specifically assessed pregnancy rates in females attempting pregnancy after diagnosis, reported pregnancy rates were comparable to our observations (77% of 26 survivors and 81% of 218 studied survivors, respectively) (Horning et al. 1981; van der Kaaij et al. 2012).

Pregnancy rates can be underestimated if participants who never had an active child wish were to be included. However, females who know they are subfertile or infertile could also indicate they have no child wish because they adjusted their future perspectives and lifegoals. Within the questionnaire, potential reasons for not wanting to become pregnant were asked. Most women felt they were still too young or reported that their current life situation (relation, study, financial, home) was not (yet) suitable for children. At the same time our questionnaire unfortunately did not include the decision making towards having children and whether having had a potential gonadotoxic treatment urged them to start their family at a younger age.

So although the probability of achieving pregnancy, resulting in a live birth, appeared to be within normal range, it should be noted that HL survivors were relatively young at time of conception when compared to the control group, and they had their first child ~ 3 years earlier than the general population (median age at first pregnancy 27 years in HL group versus 29 years in controls; Dutch population ~ 30 years old (CBS 2022)). Hypothetically, survivors were well informed about the risk of an impaired reproductive lifespan and pursued their child wish earlier than their peers. Adverse effects on pregnancy rates (or outcomes) could be more pronounced among survivors who attempt pregnancy at an older age.

In the general population, 80% of the couples conceives a pregnancy within a year (Gnoth et al. 2003; Taylor 2003; Wang et al. 2003). Several reports suggested time to conceive may be increased in cancer survivors (Barton et al. 2013; van Dijk et al. 2020). A study among childhood HL survivors, reported a median TTP of 42 months (3–100 months) among 20 women who were treated with at least five highly gonadotoxic MOPP (mechlorethamine, vincristine, prednisone, and procarbazine) courses and ~ 50% received pelvic radiation (> 3000 rads) (Horning et al. 1981). In the current study, median TTP in survivors was not as high as the previous report (4.5 months, 1–24 months) and there were no statistically significant differences observed in median TTP, compared to controls. However, adjusted analyses in which corrections were made for age at time of study, age at first pregnancy, educational level, and marital status, resulted in a significantly higher odds for having TTP > 12 months as a survivor.

Even though TTP may have been prolonged in survivors, use of assisted reproductive technology (ART) was minimal. Only 2 (4.6%) survivors and 21 (5.4%) controls achieved pregnancy via intra-uterine insemination (IUI) or in-vitro fertilization (IVF). Gonadotoxic treatment is associated with risk of impaired reserve status with fewer oocytes, but the effect on the quality of the oocytes is harder to study, so far there is no evidence demonstrating an impaired quality (Somigliana et al. 2019; van Dijk et al. 2020). Fecundability is also influenced by many other factors (such as female age, uterine function, immunology, etc.) but low AMH levels in women with an ovulatory cycle are not necessarily linked to impaired fecundity (Steiner et al. 2017; Depmann et al. 2017). Today it is unknown whether HL treatment has a lasting effect on other fertility factors explaining a prolonged time to pregnancy.

### Received HL treatment

Although the power to perform additional analyses on treatment-related factors was limited and gonadotoxicity of existing treatment protocols could not be evaluated individually, results were consistent with literature; especially after treatment with procarbazine, abnormal levels of reproductive markers were present (Drechsel et al. 2023). Analysis showed no significant effect of cyclophosphamide, although only 9 of the survivors were treated with this agent of whom 3 did not participate in the clinical assessment, meaning the power of that analysis was impaired.

A clear gonadotoxic effect of a CED-score > 6000 mg/m^2^ was observed. Our results support recent recommendations from the PanCare-LIFE consortium and IGHG guidelines-group to lower the CED cut-off from 8000 to 6000 mg/m^2^ to determine high risk treatment (Mulder et al. 2021).

It is hypothesized that the alkylating agent dacarbazine is less gonadotoxic, compared to more traditional alkylating drugs such as nitrogen mustard, cyclophosphamide, and procarbazine (Mauz-Körholz et al. 2010, 2022). The recent EuroNET-C1 study demonstrated that the substitution of dacarbazine for procarbazine in the standard HL consolidation regimen (COPP to COPDAC) reduced gonadal toxicity, with less detrimental effects on FSH levels at least one year post-treatment, without compromising event-free survival rates (Mauz-Körholz et al. 2022). In our regression analysis, we observed a potential trend towards relatively higher AMH and AFC levels after treatment with dacarbazine (corrected for other chemotherapeutics, CED-score and pelvic radiotherapy), but results did not reach statistical significance. Available fertility data is limited, and additional, long-term follow-up studies are needed to evaluate the safety of dacarbazine.

After adjusting for CED-score, no individual significant effect of abdominal radiotherapy was seen on the assessed clinical measurements. However, it is unknown if the patients who had abdominal radiation (n = 14) underwent an oophoropexy to prevent radiation effect on the ovary. Previous studies revealed that uterine radiation can negatively affect birthweight and gestational age at delivery, in a dose-dependent matter (Green et al. 2002; Sudour et al. 2010; van Dijk et al. 2020). We attempted to evaluate the effect of pelvic radiotherapy on the obstetric results of the survivor-cohort in sensitivity analyses. No statistically significant differences were observed between HL survivors who did and did not receive pelvic radiotherapy. However, presented results should be carefully interpreted due to power issues. A previous study on all CSS-subgroups of the VEVO-LATER cohort reported a clear (dose-dependent) effect of abdominal radiation on hormonal and ultrasound markers of ovarian reserve (Van Den Berg et al. 2018).

### Strengths and limitations

This study evaluated multiple clinical markers of reproductive ability and self-reported data on pregnancy outcomes in childhood HL survivors. Results were compared to a large control cohort and data cover a relatively long-time off treatment.

However, several limitations should be addressed. The studied cohort comprised a relatively young population. Risk of POI could not be assessed, and a considerable number of women indicated they considered themselves too young to aim to achieve pregnancy. Some women were still pregnant at time of questionnaire. Data were partly collected by self-report, information on for example the regularity of the menstrual cycle and TTP may not always be reliable and there may have been recall bias. Interval between clinical measurement and time of questionnaire varied widely and therefore data of both assessments could not be combined. Moreover, not all women participated in the clinical part of this study and some measurements were taken while using hormonal contraceptives. No adjustments were made to control for a potential confounding effect of polycystic ovary syndrome (PCOS) on AFC and AMH levels due to lacking data. Nevertheless, there were no statistically significant differences in cycle regularity, use of hormonal contraceptives or timing of clinical measurements between survivors and controls.

Heterogeneity in received treatment and power issues complicated the sensitivity analyses. The multivariable regression models on treatment-related factors could not be performed on dichotomized data, due to limited sample size.

### Clinical implications and future research

Survivors should be well informed about their potential risk of a reduced fertile life span after cancer treatment. Data derived from follow-up studies should be used to improve recognition of patients at high risk of adverse effects on fertility and update guidelines on fertility preservation.

Included survivors were treated between 1973 and 2001. Since then, HL regimen has evolved considerably. Most patients of the studies cohort were treated with (high doses of) procarbazine, which is completely omitted in current regimens. Study outcomes may specifically be useful to counsel HL survivors who were treated with these preceding HL regimens. There is limited data available on current HL treatment protocols and there is also a gap in knowledge on gonadal-toxicity profiles of new immunotherapeutics (e.g. Brentuximab, Nivolumab). Additional, large, prospective studies are needed to fully assess fertility after treatment for HL. Ideally, studies should have a follow-up until after the age of 40, to evaluate risk of POI and the fulfillment of the desired number of children. Multiple consecutive assessments of markers (including at least AMH and AFC) during follow-up will result in a comprehensive evaluation of reproductive ability. Logically, comparable prospective studies should be executed among male HL patients and survivors.

### Summarizing conclusions

Female HL survivors are at risk to have a reduced fertile life span. Low AMH, low AFC, elevated FSH and low inhibin B measurements were frequently present among female childhood HL survivors who were treated between 1970 and 2000s. Chance to conceive and pregnancy outcomes appear to be reassuring when attempting pregnancy at a relatively young age. Newly diagnosed patients and survivors should be counselled individually with respect to family planning and potential use of fertility preservation methods in survivorship. Additional research is needed to improve knowledge on reproductive ability after treatment of childhood HL.

### Supplementary Information

Below is the link to the electronic supplementary material.

Supplementary file1 (PDF 23 KB)

Supplementary file2 (PDF 131 KB)

Supplementary file3 (PDF 140 KB)

Supplementary file4 (PDF 231 KB)

## Data Availability

The authors confirm that the data supporting the findings of this study are available within the article and its supplementary materials.

## References

[CR1] Absolom K, Eiser C, Turner L, Ledger W, Ross R, Davies H, Coleman R, Hancock B, Snowden J, Greenfield D, Sheffield, on behalf of the LEG (2008). Ovarian failure following cancer treatment: current management and quality of life. Hum Reprod.

[CR2] Anderson RA, Su HI (2020). The clinical value and interpretation of anti-müllerian hormone in women with cancer. Front Endocrinol.

[CR3] Anderson RA, Brewster DH, Wood R, Nowell S, Fischbacher C, Kelsey TW, Wallace WHB (2018). The impact of cancer on subsequent chance of pregnancy: a population-based analysis. Hum Reprod.

[CR4] Anderson RA, Cameron D, Clatot F, Demeestere I, Lambertini M, Nelson SM, Peccatori F (2022). Anti-Müllerian hormone as a marker of ovarian reserve and premature ovarian insufficiency in children and women with cancer: a systematic review. Hum Reprod Update.

[CR5] Anderson RA, Kelsey TW, Morrison DS, Wallace WHB (2022). Family size and duration of fertility in female cancer survivors: a population-based analysis. Fertil Steril.

[CR6] Armuand G, Skoog-Svanberg A, Bladh M, Sydsjö G (2017). Reproductive patterns among childhood and adolescent cancer survivors in Sweden: a population-based matched-cohort study. J Clin Oncol.

[CR7] Barton SE, Najita JS, Ginsburg ES, Leisenring WM, Stovall M, Weathers RE, Sklar CA, Robison LL, Diller L (2013). Infertility, infertility treatment, and achievement of pregnancy in female survivors of childhood cancer: a report from the Childhood Cancer Survivor Study cohort. Lancet Oncol.

[CR8] Bines J, Oleske DM, Cobleigh MA (1996). Ovarian function in premenopausal women treated with adjuvant chemotherapy for breast cancer. J Clin Oncol.

[CR9] Borchmann P, Eichenauer DA, Engert A (2012). State of the art in the treatment of Hodgkin lymphoma. Nat Rev Clin Oncol.

[CR10] Brämswig JH, Riepenhausen M, Schellong G (2015). Parenthood in adult female survivors treated for Hodgkin’s lymphoma during childhood and adolescence: a prospective, longitudinal study. Lancet Oncol.

[CR11] Broekmans FJ, Soules MR, Fauser BC (2009). Ovarian aging: mechanisms and clinical consequences. Endocr Rev.

[CR12] Broer SL, Broekmans FJM, Laven JSE, Fauser BCJM (2014). Anti-Müllerian hormone: ovarian reserve testing and its potential clinical implications. Hum Reprod Update.

[CR13] CBS (2022) Statline: Geboorte; kerncijfers. https://www.cbs.nl/nl-nl/cijfers/detail/37422ned Accessed 4 May 2023

[CR14] Chapman RM (1982) Effect of cytotoxic therapy on sexuality and gonadal function. Semin Oncol 9(1), 84–94. 6176028

[CR15] Charpentier AM, Chong AL, Gingras-Hill G, Ahmed S, Cigsar C, Gupta AA, Greenblatt E, Hodgson DC (2014). Anti-Müllerian hormone screening to assess ovarian reserve among female survivors of childhood cancer. J Cancer Surviv.

[CR16] Chow EJ, Stratton KL, Leisenring WM, Oeffinger KC, Sklar CA, Donaldson SS, Ginsberg JP, Kenney LB, Levine JM, Robison LL (2016). Pregnancy after chemotherapy in male and female survivors of childhood cancer treated between 1970 and 1999: a report from the Childhood Cancer Survivor Study cohort. Lancet Oncol.

[CR17] De Vos M, Devroey P, Fauser BC (2010). Primary ovarian insufficiency. The Lancet.

[CR18] Depmann M, Broer SL, Eijkemans MJC, van Rooij IAJ, Scheffer GJ, Heimensem J, Mol BW, Broekmans FJM (2017). Anti-Müllerian hormone does not predict time to pregnancy: results of a prospective cohort study. Gynecolog Endocrinol.

[CR19] Drechsel KCE, Pilon MCF, Stoutjesdijk F, Meivis S, Schoonmade LJ, Wallace WHB, van Dulmenden Broeder E, Beishuizen A, Kaspers GJL, Broer SL, Veening MA (2023) Reproductive ability in survivors of childhood, adolescent, and young adult Hodgkin lymphoma: a review. Human reproduction update 29(4): 486–517 10.1093/humupd/dmad002 10.1093/humupd/dmad002 PMC10320502 36779325

[CR20] European Network-Paediatric Hodgkin Lymphoma Study Group (EuroNet-PHL) (2015) Second international Inter-Group Study for Classical Hodgkin Lymphoma in Children and Adolescent: EuroNet-PHL-C2

[CR21] Fernandez-Pineda I, Davidoff AM, Lu L, Rao BN, Wilson CL, Srivastava DK, Klosky JL, Metzger ML, Krasin MJ, Ness KK, Pui CH, Robison LL, Hudson MM, Sklar CA, Green DM, Chemaitilly W (2018). Impact of ovarian transposition before pelvic irradiation on ovarian function among long-term survivors of childhood Hodgkin lymphoma: a report from the St Jude Lifetime Cohort Study. Pediatr Blood Cancer.

[CR22] Fleming R, Kelsey TW, Anderson RA, Wallace WH, Nelson SM (2012). Interpreting human follicular recruitment and antimüllerian hormone concentrations throughout life. Fertil Steril.

[CR23] Gnoth C, Godehardt D, Godehardt E, Frank-Herrmann P, Freundl G (2003). Time to pregnancy: results of the German prospective study and impact on the management of infertility. Hum Reprod.

[CR24] Gözdasoglu S, Cavdar AO, Babacan E, Mengübas K, Yavuz G, Unal E, Pamir A, Ocal G, Haluk Gökçora I (1995). Late effects of chemoradiotherapy in pediatric Hodgkin’s disease. J Chemother.

[CR25] Green DM, Hall B (1988). Pregnancy outcome following treatment during childhood or adolescence for Hodgkin’s disease. Pediatr Hematol Oncol.

[CR26] Green DM, Whitton JA, Stovall M, Mertens AC, Donaldson SS, Ruymann FB, Pendergrass TW, Robison LL (2002). Pregnancy outcome of female survivors of childhood cancer: a report from the Childhood Cancer Survivor Study. Am J Obstet Gynecol.

[CR27] Green DM, Kawashima T, Stovall M, Leisenring W, Sklar CA, Mertens AC, Donaldson SS, Byrne J, Robison LL (2009). Fertility of female survivors of childhood cancer: a report from the childhood cancer survivor study. J Clin Oncol.

[CR28] Green DM, Nolan VG, Goodman PJ, Whitton JA, Srivastava DK, Leisenring WM, Neglia JP, Sklar CA, Kaste SC, Hudson MM, Diller LR, Stovall M, Donaldson SS, Robison LL (2014). The cyclophosphamide equivalent dose as an approach for quantifying alkylating agent exposure: A report from the childhood cancer survivor study. Pediatric Blood.

[CR29] Horning SJ, Hoppe RT, Kaplan HS, Rosenberg SA (1981). Female reproductive potential after treatment for Hodgkin’s disease. N Engl J Med.

[CR30] Irene Su H, Kwan B, Whitcomb BW, Shliakhsitsava K, Dietz AC, Stark SS, Martinez E, Sluss PM, Sammel MD, Natarajan L (2020). Modeling variation in the reproductive lifespan of female adolescent and young adult cancer survivors using AMH. J Clin Endocrinol Metab.

[CR31] Jiao X, Meng T, Zhai Y, Zhao L, Luo W, Liu P, Qin Y (2021). Ovarian reserve markers in premature ovarian insufficiency: within different clinical stages and different etiologies. Front Endocrinol.

[CR32] Kelsey TW, Wright P, Nelson SM, Anderson RA, Wallace WHB (2011). A validated model of serum Anti-Müllerian hormone from conception to menopause. PLoS ONE.

[CR33] Kim CH, Jeon GH (2012) Fertility preservation in female cancer patients. ISRN obstetrics and gynecology, 2012, 807302. 10.5402/2012/807302 10.5402/2012/807302 PMC3302115 22462006

[CR34] Krawczuk-Rybak M, Leszczynska E, Poznanska M, Zelazowska-Rutkowska B, Wysocka J (2013). Anti-Müllerian hormone as a sensitive marker of ovarian function in young cancer survivors. Int J Endocrinol.

[CR35] Landier W, Skinner R, Wallace WH, Hjorth L, Mulder RL, Wong FL, Yasui Y, Bhakta N, Constine LS, Bhatia S, Kremer LC, Hudson MM (2018). Surveillance for late effects in childhood cancer survivors. J Clin Oncol.

[CR36] Laporte S, Couto-Silva A-C, Trabado S, Lemaire P, Brailly-Tabard S, Espérou H, Michon J, Baruchel A, Fischer A, Trivin C, Brauner R (2011). Inhibin B and anti-Müllerian hormone as markers of gonadal function after hematopoietic cell transplantation during childhood. BMC Pediatr.

[CR37] Licht SF, Rugbjerg K, Andersen EW, Nielsen TT, Norsker FN, Kenborg L, Holmqvist AS, Madanat-Harjuoja LM, Tryggvadottir L, Stovall M, Wesenberg F, Hjorth L, Hasle H, Winther JF (2021). Temporal changes in the probability of live birth among female survivors of childhood cancer: a population-based Adult Life After Childhood Cancer in Scandinavia (ALiCCS) study in five nordic countries. Cancer.

[CR38] Mackie EJ, Radford M, Shalet SM (1996). Gonadal function following chemotherapy for childhood Hodgkin’s disease. Med Pediatr Oncol.

[CR39] Madanat LS, Malila N, Dyba T, Hakulinen T, Sankila R, Boice JD, Lähteenmäki PM (2008). Probability of parenthood after early onset cancer: a population-based study. Int J Cancer.

[CR40] Madanat-Harjuoja L, Malila N, Lähteenmäki PM, Boice JD, Gissler M, Dyba T (2010). Preterm delivery among female survivors of childhood, adolescent and young adulthood cancer. Int J Cancer.

[CR41] Madsen BL, Giudice L, Donaldson SS (1995). Radiation-induced premature menopause: a misconception. Int J Radiat Oncol Biol Phys.

[CR42] Magelssen H, Melve KK, Skjaerven R, Fosså SD (2008). Parenthood probability and pregnancy outcome in patients with a cancer diagnosis during adolescence and young adulthood. Hum Reprod.

[CR43] Mauz-Körholz C, Hasenclever D, Dörffel W, Ruschke K, Pelz T, Voigt A, Stiefel M, Winkler M, Vilser C, Dieckmann K, Karlén J, Bergsträsser E, Fosså A, Mann G, Hummel M, Klapper W, Stein H, Vordermark D, Kluge R, Körholz D (2010). Procarbazine-free OEPA-COPDAC chemotherapy in boys and standard OPPA-COPP in girls have comparable effectiveness in pediatric Hodgkin’s lymphoma: The GPOH-HD-2002 study. J Clin Oncol.

[CR44] Mauz-Körholz C, Landman-Parker J, Balwierz W, Ammann RA, Anderson RA, Attarbaschi A, Bartelt JM, Beishuizen A, Boudjemaa S, Cepelova M, Claviez A, Daw S, Dieckmann K, Fernández-Teijeiro A, Fosså A, Gattenlöhner S, Georgi T, Hjalgrim LL, Hraskova A, Wallace WH (2022). Response-adapted omission of radiotherapy and comparison of consolidation chemotherapy in children and adolescents with intermediate-stage and advanced-stage classical Hodgkin lymphoma (EuroNet-PHL-C1): a titration study with an open-label, embedded, multinational, non-inferiority, randomised controlled trial. Lancet Oncol.

[CR45] McCullough L, Ng A, Najita J, Janov A, Henderson T, Mauch P, Diller L (2010). Breastfeeding in survivors of Hodgkin lymphoma treated with chest radiotherapy. Cancer.

[CR46] Morabia A (1998). International variability in ages at menarche, first livebirth, and menopause. Am J Epidemiol.

[CR47] Mulder RL, Font-Gonzalez A, Hudson MM, van Santen HM, Loeffen EAH, Burns KC, Quinn GP, van Dulmen-den Broeder E, Byrne J, Haupt R, Wallace WH, van den Heuvel-Eibrink MM, Anazodo A, Anderson RA, Barnbrock A, Beck JD, Bos AME, Demeestere I, Denzer C, Verhaak C (2021). Fertility preservation for female patients with childhood, adolescent, and young adult cancer: recommendations from the PanCareLIFE Consortium and the International Late Effects of Childhood Cancer Guideline Harmonization Group. Lancet Oncol.

[CR48] Oktem O, Kim SS, Selek U, Schatmann G, Urman B (2018). Ovarian and uterine functions in female survivors of childhood cancers. Oncologist.

[CR49] Overbeek A, van den Berg MH, Kremer LCM, van den Heuvel-Eibrink MM, Tissing WJE, Loonen JJ, Versluys B, Bresters D, Kaspers GJL, Lambalk CB, van Leeuwen FE, van Dulmen-den Broeder E, Beerendonk CCM, Bökkerink JP, van den Bos C, van Dorp W, van Engelen MP, Huizinga GA, Jaspers MWM, Verkerk ECM (2012). A nationwide study on reproductive function, ovarian reserve, and risk of premature menopause in female survivors of childhood cancer: design and methodological challenges. BMC.

[CR50] Papadakis V, Vlachopapadopoulou E, Van Syckle K, Ganshaw L, Kalmanti M, Tan C, Sklar C (1999). Gonadal function in young patients successfully treated for Hodgkin disease. Med Pediatr Oncol.

[CR51] Parry JP, Koch CA (2019) Ovarian reserve testing. Endotext [Internet].

[CR52] Perrone L, Sinisi AA, Di Tullio MT, Casale F, Indolfi P, Manzo T, Coppola G, Bellastella A, Faggiano M (1989). Endocrine function in subjects treated for childhood Hodgkins’ disease. J Pediatr Endocrinol Metab.

[CR53] R Core Team (2018). R: a language and environment for statistical computing.

[CR54] Reulen RC, Zeegers MP, Wallace WHB, Frobisher C, Taylor AJ, Lancashire ER, Winter DL, Hawkins MM (2009). Pregnancy outcomes among adult survivors of childhood cancer in the British childhood cancer survivor study. Cancer Epidemiol Biomark Prev.

[CR55] Reulen RC, Bright CJ, Winter DL, Fidler MM, Wong K, Guha J, Kelly JS, Frobisher C, Edgar AB, Skinner R, Wallace WHB, Hawkins MM (2017). Pregnancy and labor complications in female survivors of childhood cancer: the british childhood cancer survivor study. J Natl Cancer Inst.

[CR56] Robison LL, Hudson MM (2014). Survivors of childhood and adolescent cancer: life-long risks and responsibilities. Nat Rev Cancer.

[CR57] Rodriguez-Wallberg KA, Anastacio A, Vonheim E, Deen S, Malmros J, Borgström B (2020). Fertility preservation for young adults, adolescents, and children with cancer. Upsala J Med Sci.

[CR58] Roshandel R, van Dijk M, Overbeek A, Kaspers G, Lambalk C, Beerendonk C, Bresters D, van der Heiden-van der Loo M, van den Heuvel-Eibrink M, Kremer L, Loonen J, van der Pal H, Ronckers C, Tissing W, Versluys B, van Leeuwen F, van den Berg M, van Dulmen-den Broeder E (2021) Female reproductive function after treatment of childhood acute lymphoblastic leukemia. Pediatric Blood Cancer 68(4). 10.1002/pbc.28894 10.1002/pbc.28894 33459500

[CR59] Siegel RL, Miller KD, Wagle NS, Jemal A (2023). Cancer statistics, 2023. CA Cancer J Clin.

[CR60] Somigliana E, Terenziani M, Filippi F, Bergamini A, Martinelli F, Mangili G, Peccatori F, Vercellini P (2019). Chemotherapy-related damage to ovarian reserve in childhood cancer survivors: interpreting the evidence. J Assist Reprod Genet.

[CR61] Steiner AZ, Pritchard D, Stanczyk FZ, Kesner JS, Meadows JW, Herring AH, Baird DD (2017). Association between biomarkers of ovarian reserve and infertility among older women of reproductive age. JAMA.

[CR62] Sudour H, Chastagner P, Claude L, Desandes E, Klein M, Carrie C, Bernier V (2010). Fertility and pregnancy outcome after abdominal irradiation that included or excluded the pelvis in childhood tumor survivors. Int J Radiat Oncol Biol Phys.

[CR63] Swerdlow AJ, Jacobs PA, Marks A, Maher EJ, Young T, Barber JC, Vaughan Hudson G (1996). Fertility, reproductive outcomes, and health of offspring, of patients treated for Hodgkin’s disease: an investigation including chromosome examinations. Br J Cancer.

[CR64] Sy Ortin TT, Shostak CA, Donaldson SS (1990). Gonadal status and reproductive function following treatment for Hodgkin’s disease in childhood: the Stanford experience. Int J Radiat Oncol Biol Phys.

[CR65] Taylor A (2003). Extent of the Problem. BMJ.

[CR66] Te Velde ER, Pearson PL (2002). The variability of female reproductive ageing. Hum Reprod Update.

[CR67] Van Beek RD, Van Den Heuvel-eibrink MM, Laven JSE, De Jong FH, Themmen APN, Hakvoort-Cammel FG, Bos CV, Den B, Van Den H, Pieters R, Keizer-schrama SMPFDM, van Beek RD, van den Heuvel-Eibrink MM, Laven JSE, de Jong FH, Themmen APN, Hakvoort-Cammel FG, van den Bos C, van den Berg H, Pieters R, de Muinck Keizer-Schrama SM (2007). Anti-Mullerian hormone is a sensitive serum marker for gonadal function in women treated for Hodgkin’s lymphoma during childhood. J Clin Endocrinol Metab.

[CR68] van de Loo LEXM, van den Berg MH, Overbeek A, van Dijk M, Damen L, Lambalk CB, Ronckers CM, van den Heuvel-Eibrink MM, Kremer, LCM, van der Pal HJ, Laven JSE, Tissing WJE, Loonen JJ, Versluys B, Bresters D, Kaspers GJL, van Leeuwen FE, van Dulmen-den Broeder E (2019). Uterine function, pregnancy complications, and pregnancy outcomes among female childhood cancer survivors. FertilSteril 111(2): 372–380. 10.1016/j.fertnstert.2018.10.016 10.1016/j.fertnstert.2018.10.016 30691634

[CR69] Van Den Berg MH, Van Dulmen-Den Broeder E, Overbeek A, Ronckers CM, Van Dorp W, Kremer LC, Van Den Heuvel-Eibrink MM, Huizinga GA, Loonen JJ, Versluys AB, Bresters D, Lambalk CB, Kaspers GJL, Van Leeuwen FE (2014). Fertility studies in female childhood cancer survivors: Selecting appropriate comparison groups. Reprod Biomed Online.

[CR70] Van Den Berg MH, Overbeek A, Lambalk CB, Kaspers GJL, Bresters D, Van Den Heuvel-Eibrink MM, Kremer LC, Loonen JJ, Van Der Pal HJ, Ronckers CM, Tissing WJE, Versluys AB, Van Der Heiden-Van Der Loo M, Heijboer AC, Hauptmann M, Twisk JWR, Laven JSE, Beerendonk CCM, Van Leeuwen FE, Van Dulmen-Den Broeder E (2018) Long-term effects of childhood cancer treatment on hormonal and ultrasound markers of ovarian reserve. Human Reprod 33(8): 1474–1488. 10.1093/humrep/dey229 10.1093/humrep/dey229 29982673

[CR71] van den Berg MH, van Dijk M, Byrne J, Berger C, Dirksen U, Winther JF, Fossa SD, Grabow D, Grandage VL, Haupt R, van den Heuvel-Eibrink MM, Kaiser M, Kepak T, van der Kooi ALF, Kremer LCM, Kruseova J, Lambalk CB, van Leeuwen FE, Leiper A, Baust K (2021) Treatment-related fertility impairment in long-term female childhood, adolescent and young adult cancer survivors: investigating dose-effect relationships in a European case-control study (PanCareLIFE). Huma Reprod 36(6), 1561–1573. 10.1093/humrep/deab035 10.1093/humrep/deab035 33744927

[CR72] van der Kaaij MA, Heutte N, Meijnders P, Abeilard-Lemoisson E, Spina M, Moser LC, Allgeier A, Meulemans B, Dubois B, Simons AH, Lugtenburg PJ, Aleman BM, Noordijk EM, Fermé C, Thomas J, Stamatoullas A, Fruchart C, Brice P, Gaillard I, Kluin-Nelemans HC (2012). Parenthood in survivors of Hodgkin lymphoma: an EORTC-GELA general population case-control study. J Clin Oncol.

[CR73] Van Der Kooi ALF, Van Den Heuvel-Eibrink MM, Van Noortwijk A, Neggers SJCMM, Pluijm SMF, Van Dulmen-Den Broeder E, Van Dorp W, Laven JSE (2017). Longitudinal follow-up in female Childhood Cancer Survivors: no signs of accelerated ovarian function loss. Hum Reprod.

[CR74] van Dijk M, van Leeuwen FE, Overbeek A, Lambalk CB, van den Heuvel-Eibrink MM, van Dorp W, Tissing WJ, Kremer LC, Loonen JJ, Versluys B, Bresters D, Ronckers CM, van der Pal HJ, Beerendonk CCM, Kaspers GJL, van Dulmen-den Broeder E, van den Berg MH (2020). Pregnancy, time to pregnancy and obstetric outcomes among female childhood cancer survivors: results of the DCOG LATER-VEVO study. J Res Clin Oncol.

[CR75] Visser GHA, Eilers PHC, Elferink-Stinkens PM, Merkus HMWM, Wit JM (2009). New Dutch reference curves for birthweight by gestational age. Early Human Dev.

[CR76] Wang X, Chen C, Wang L, Chen D, Guang W, French J, Group RHS (2003). Conception, early pregnancy loss, and time to clinical pregnancy: a population-based prospective study. Fertil Steril.

[CR77] Webber L, Davies M, Anderson R, Bartlett J, Braat D, Cartwright B, Cifkova R, De Muinck Keizer-Schrama S, Hogervorst E, Janse F, Liao L, Vlaisavljevic V, Zillikens C, Vermeulen N (2016). ESHRE Guideline: management of women with premature ovarian insufficiency. Hum Reprod.

[CR78] Wesselink AK, Rothman KJ, Hatch EE, Mikkelsen EM, Sørensen HT, Wise LA (2017). Age and fecundability in a North American preconception cohort study. Am J Obstet Gynecol.

[CR79] Wilimas J, Thompson E, Smith KL (1980). Long-term results of treatment of children and adolescents with Hodgkin’s disease. Cancer.

