# Supplementary material for: Clinical and self-reported markers of reproductive function in female survivors of childhood Hodgkin lymphoma
Source: J Cancer Res Clin Oncol. 2023 Jul 31;149(15):13677–95. doi: 10.1007/s00432-023-05035-z (PMC10590326; doi:10.1007/s00432-023-05035-z)
Supplement: Supplementary file 1 — Supplementary file1 (PDF 23 KB) [file 432_2023_5035_MOESM1_ESM.pdf]

Online resource 1

Supplementary table: Combinations of abnormal clinical markers of reproductive function in female childhood or adolescent Hodgkin lymphoma survivors

Clinical measurements (combined)	Clinical measurement (per measurement)				HL survivor *(n=39)*	Controls *(n=340)*
	AMH	AFC	FSH	Inhibin B		
All normal	-	-	-	-	18 (46.2%)	253 (74.4%)
All abnormal	**low**	**low**	**high**	**low**	8 (20.5%)	4 (1.2%)
Low AMH, Normal AFC, FSH, Inhibin	**low**	-	-	-	6 (15.4%)	9 (2.6%)
Low AMH and high FSH, Normal AFC and inhibin B	**low**	-	**high**	-	3 (7.7%)	3 (0.9%)
Low AMH and low inhibin B, Normal AFC and FSH	**low**	-	-	**low**	2 (5.1%)	7 (2.1%)
High FSH and Low inhibin B, Normal AMH and AFC	-	-	**high**	**low**	2 (5.1%)	18 (5.3%)
High FSH, normal AMH, AFC, Inhibin B	-	-	**high**	-	-	16 (4.7%)
Low inhibin B, normal AMH, AFC, FSH	-	-	-	**low**	-	30 (8.8%)

- laboratory measurements were within normal limits.
AMH; anti-mullerian hormone, AFC; antral follicle count, FSH; follicle stimulating hormone, HL; Hodgkin lymphoma

Applied cut-off values to determine low/high values: AMH <p10 of healthy controls, AFC <p10 of healthy controls, FSH >10IU/L, Inhibin B <20 IU/L

Article title: Clinical and self-reported markers of reproductive function in female survivors of childhood Hodgkin lymphoma
Journal: Journal of Cancer Research and Clinical Oncology
Authors: K.C.E. Drechsel*, S.L. Broer, F. Stoutjesdijk, J.W.R. Twisk, M.H. van den Berg, C.B. Lambalk, F.E. van Leeuwen, A. Overbeek, M.M. van den Heuvel-Eibrink, W. van Dorp, A.C.H. de Vries, J.J. Loonen, H.J. van der Pal, L.C. Kremer, W.J. Tissing, B. Versluys, G.J.L. Kaspers, E. van Dulmen-den Broeder**, M.A. Veening**
on behalf of the LATER-VEVO study group.
***shared last authorship*

***Corresponding author:**
Drs. K.C.E. Drechsel, MD
Pediatric Oncology, Emma Children's Hospital, Amsterdam UMC, Vrije Universiteit Amsterdam, Amsterdam, The Netherlands.
Princess Máxima Centre for Pediatric Oncology, 3584 CS Utrecht, The Netherlands.
Cancer Center Amsterdam, Amsterdam UMC, location VUmc, VU Amsterdam, 1007 MB Amsterdam, Netherlands.
k.c.e.drechsel@amsterdamumc.nl / ORCHID iD: 0000-0001-9879-4678
