# Supplementary material for: Clinical and self-reported markers of reproductive function in female survivors of childhood Hodgkin lymphoma
Source: J Cancer Res Clin Oncol. 2023 Jul 31;149(15):13677–95. doi: 10.1007/s00432-023-05035-z (PMC10590326; doi:10.1007/s00432-023-05035-z)
Supplement: Supplementary file 2 — Supplementary file2 (PDF 131 KB) [file 432_2023_5035_MOESM2_ESM.pdf]

Online resource 2

Supplementary Table: Additional information on childhood Hodgkin lymphoma survivors and controls who did not attempt pregnancy

		HL survivors (n=37)	Controls (n=368)
**Age**	Median (range)	25.6 [18.6;55.8]	26.6 [18.0;51.3]
	18-25	16 (43.2%)	148 (40.2%)
	25-30	15 (40.5%)	107 (29.1%)
	30-35	4 (10.8%)	65 (17.7%)
	35-40	1 (2.7%)	32 (8.7%)
	>40	1 (2.7%)	16 (4.3%)
**Marital status**	Single	12 (32.4%)	124 (33.7%)
	Married/relationship	25 (67.6%)	239 (64.9%)
	Divorced/widowed	0 (0%)	1 (0.3%)
**Educational level**	Low	0 (0%)	4 (1.1%)
	Medium	13 (35.1%)	116 (31.5%)
	High	24 (64.9%)	245 (66.6%)
**Reason to have never attempted pregnancy (multiple answers possible)**	No partner	13 (35.1%)*	101 (27.4%)
	Not ready (yet)	19 (51.4%)	217 (59.0%)
	My partner is not (yet) ready	4 (10.8%)	79 (21.5%)
	I never wanted to have kids	1 (2.7%)	28 (7.6%)
	I don't know yet, have not thought about it (enough)	2 (5.4%)	13 (3.5%)
	I don't know yet, because of my medical history	2 (5.4%)	2 (0.5%)
	(other) personal reasons	1 (2.7%)	4 (1.1%)
	Did not answer	0 (0%)	22 (5.9%)
	Other, mentioned reasons were about study, career, age, and/or health of partner	4 (10.8%)	47 (12.8%)

HL; Hodgkin lymphoma
*Marital status is single in n=12 survivors according to the questionnaire data, while n=13 participants mentioned having no partner is a reason for not attempting pregnancy. These results are re-checked and in line with actual response data.

Article title: Clinical and self-reported markers of reproductive function in female survivors of childhood Hodgkin lymphoma
Journal: Journal of Cancer Research and Clinical Oncology
Authors: K.C.E. Drechsel*, S.L. Broer, F. Stoutjesdijk, J.W.R. Twisk, M.H. van den Berg, C.B. Lambalk, F.E. van Leeuwen, A. Overbeek, M.M. van den Heuvel-Eibrink, W. van Dorp, A.C.H. de Vries, J.J. Loonen, H.J. van der Pal, L.C. Kremer, W.J. Tissing, B. Versluys, G.J.L. Kaspers, E. van Dulmen-den Broeder**, M.A. Veening**
on behalf of the LATER-VEVO study group.
***shared last authorship*

***Corresponding author:**
Drs. K.C.E. Drechsel, MD
Pediatric Oncology, Emma Children's Hospital, Amsterdam UMC, Vrije Universiteit Amsterdam, Amsterdam, The Netherlands.
Princess Máxima Centre for Pediatric Oncology, 3584 CS Utrecht, The Netherlands.
Cancer Center Amsterdam, Amsterdam UMC, location VUmc, VU Amsterdam, 1007 MB Amsterdam, Netherlands.
k.c.e.drechsel@amsterdamumc.nl / ORCHID iD: 0000-0001-9879-4678
