# Supplementary material for: Clinical and self-reported markers of reproductive function in female survivors of childhood Hodgkin lymphoma
Source: J Cancer Res Clin Oncol. 2023 Jul 31;149(15):13677–95. doi: 10.1007/s00432-023-05035-z (PMC10590326; doi:10.1007/s00432-023-05035-z)
Supplement: Supplementary file 4 — Supplementary file4 (PDF 231 KB) [file 432_2023_5035_MOESM4_ESM.pdf]

Online resource 4

Supplementary Table: First pregnancy in Hodgkin lymphoma survivors, comparing received treatment with CED-score >6000mg/m2 and ≤6000mg/m2.

		CED-score ≤6000 mg/m2	CED-score >6000 mg/m2	P value
		24	*56*	
**Ever attempted to become pregnant**	n (%)	10 (41.7%)	34 (60.7%)	0.185
		10	*34*	
**Ever pregnant**	n (%)	9 (90.0%)	32 (94.1%)	0.548
**Ever achieved a live birth**	n (%)	7 (70.0%)	30 (88.2%)	0.322
**Ever miscarried**	n (%)	1 (10.0%)	8 (23.5%)	0.659
**Known outcomes for (first) pregnancies within cohort**		*9*	*32*	
**Age at first pregnancy (years)**	Median (IQR)	28.0 [21.8;34.1]	27.5 [21.0;35.7]	0.528
**Currently pregnant (first pregnancy)**	n(%)	2 (22.22%)	1 (3.1%)	0.116
**Live birth**	n (%)	6 (66.7%)	27 (84.4%)	0.342
**Still birth**	n (%)	0	0	-
**Miscarriage**	n (%)	1 (11.1%)	2 (6.2%)	0.535
**APLA**	n (%)	0	2 (6.2%)	
**Ectopic pregnancy**	n (%)	0	0	
**Time to pregnancy (TTP) (months)**	Median (IQR)	5.0 [1.0;22.6]	5.5 [1.0;33.0]	1.000
	n (%) with TTP >12 months	2 (25.0%)	8 (25.0%)	1.000
**Use of ART**	n (%)	0	2 (6.2%)	1.000
	- IUI	0	1 (3.1%)	1.000
	- IVF/ICSI	0	1 (3.1%)	1.000
Obstetric outcomes				
**Birth weight (gram)**	Median (IQR)	3435.0 [3006.9;4512.5]	3174.5 [1552.5;4288.5]	0.277
**Small for gestational age (SGA)**	n (%)	0 (0.0%)	5 (15.4%)	0.566
**GA at delivery**	Median (IQR)	39.5 [37.0;41.9]	40.0 [31.3;42.0]	0.774
**Preterm delivery**	n (%)	0 (0.0%)	3 (11.1%)	1.000

CED-score: cyclophosphamide equivalent dose
Differences were analyzed using chi-square test or Fisher's exact tests for categorical variables and Mann–Whitney U-test for continuous variables (because of non-normal distribution).

Article title: Clinical and self-reported markers of reproductive function in female survivors of childhood Hodgkin lymphoma
Journal: Journal of Cancer Research and Clinical Oncology
Authors: K.C.E. Drechsel*, S.L. Broer, F. Stoutjesdijk, J.W.R. Twisk, M.H. van den Berg, C.B. Lambalk, F.E. van Leeuwen, A. Overbeek, M.M. van den Heuvel-Eibrink, W. van Dorp, A.C.H. de Vries, J.J. Loonen, H.J. van der Pal, L.C. Kremer, W.J. Tissing, B. Versluys, G.J.L. Kaspers, E. van Dulmen-den Broeder**, M.A. Veening**
on behalf of the LATER-VEVO study group.
***shared last authorship*

***Corresponding author:**
Drs. K.C.E. Drechsel, MD
Pediatric Oncology, Emma Children's Hospital, Amsterdam UMC, Vrije Universiteit Amsterdam, Amsterdam, The Netherlands.

Princess Máxima Centre for Pediatric Oncology, 3584 CS Utrecht, The Netherlands.
Cancer Center Amsterdam, Amsterdam UMC, location VUmc, VU Amsterdam, 1007 MB Amsterdam, Netherlands.
k.c.e.drechsel@amsterdamumc.nl / ORCHID iD: 0000-0001-9879-4678
